# Efficacy of fluoride associated with nano-hydroxyapatite in reducing enamel demineralization adjacent to orthodontic brackets: in situ study

**DOI:** 10.1590/2177-6709.24.6.048-055.oar

**Published:** 2019

**Authors:** Carina Faleiros Demito, Julyano Vieira da Costa, Marina de Lourdes Calvo Fracasso, Adilson Luiz Ramos

**Affiliations:** 1 Universidade Estadual de Maringá (Maringá/PR, Brazil).; 2 Universidade Estadual de Maringá, Departamento de Odontologia Integrada (Maringá/PR, Brazil).; 3 Universidade de São Paulo, Departamento de Odontopediatria (Bauru/SP, Brazil).; 4 Universidade Estadual de Maringá, Departamento de Odontologia (Maringá/PR, Brazil).

**Keywords:** Tooth demineralization, Nano-hydroxyapatite, Fluorides, Dental caries, Orthodontic treatment

## Abstract

**Objective:**

To assess *in situ* the effect of fluoride associated with nano-hydroxyapatite for the prevention of demineralization of the enamel adjacent to orthodontic brackets.

**Material and Methods:**

Eight volunteers wore palatal devices prepared with 6 bovine enamel blocks (5*x*5*x*2 mm) with bonded brackets. The volunteers used the devices in two different moments of 14 days each. During the first 14 days, a product containing fluoride + nano-hydroxyapatite was applied twice (experimental group, GNH, n = 48), and for the other 14 days no prevention product was applied (control group, CG, n = 48). In both groups, along the experiment, the blocks were dripped with 20% sucrose eight times daily. After the experiment, all the specimens were sectioned and examined for lesion depth analysis (µm) under polarized light microscopy, and for enamel longitudinal microhardness (measured under the bracket, at 30 µm and at 130 µm from the margin), at seven different depths (10, 20, 30, 50, 70, 90, and 110 µm).

**Results:**

Under polarized light, group GNH presented significantly less demineralization depth ($\bar{X}$= 15.01 µm, SD = 33.65) in relation to CG ($\bar{X}$= 76.43 µm, SD = 83.75). Enamel longitudinal microhardness demonstrated significantly higher microhardness for group GNH when compared to CG.

**Conclusion:**

Fluoride + nano-hydroxyapatite can be an alternative preventive procedure for demineralization of the enamel adjacent to orthodontic brackets.

## RESUMO

**Objetivo:**

avaliar *in situ* o efeito da nano-hidroxiapatita associada ao fluoreto na prevenção da desmineralização do esmalte adjacente aos braquetes ortodônticos.

**Material e Métodos:**

oito voluntários utilizaram aparelhos palatinos preparados com seis blocos de esmalte bovino (5 x 5 x 2 mm) com braquetes colados unidos por um fio de NiTi 0,016” preso por ligaduras elásticas. Os voluntários utilizaram os dispositivos em dois tempos distintos de 14 dias. Durante os primeiros 14 dias, um produto contendo flúor + nano-hidroxiapatita foi aplicado duas vezes (grupo experimental, GNH, n = 48), e nos outros 14 dias não foi aplicado qualquer produto de prevenção (grupo controle, GC, n = 48). Em ambos os grupos, ao longo do experimento, os blocos foram gotejados com sacarose a 20%, 8 vezes ao dia. Após o experimento, todos os espécimes foram seccionados e examinados para análise de profundidade de lesão (µm), sob microscopia de luz polarizada, e para microdureza longitudinal do esmalte do braquete (a 30µm e a 130µm da margem), a 7 profundidades diferentes (10, 20, 30, 50, 70, 90 e 110µm)

**Resultados:**

sob luz polarizada, o grupo GNH apresentou significativamente menor profundidade de desmineralização ($\bar{X}$= 15,01µm, D.P. = 33,65) em relação ao GC ($\bar{X}$= 76,43µm, D.P. = 83,75) A microdureza longitudinal do esmalte demonstrou valor significativamente maior para o grupo GNH, quando comparado ao GC.

**Conclusão:**

a nano-hidroxiapatita associada ao fluoreto pode ser um procedimento alternativo para a prevenção da desmineralização do esmalte adjacente aos braquetes ortodônticos.

## INTRODUCTION

Enamel demineralization takes place in almost 50% of orthodontic patients treated with fixed appliances^1^ ^-^ ^5^. This is especially due to deficient oral hygiene by the patient. A fact that is aggravated by the increased retentivity of bacterial plaque around the brackets, which may be two or three times higher than in patients without fixed appliances^6^ ^,^ ^7^. The surfaces of teeth that normally are less susceptible to caries as, for instance, the buccal surfaces, become the target for the development of such lesions, especially at the gingival region.^3^

Despite the advances in caries prevention techniques, preventing demineralization during orthodontic treatments continues to be a challenge for the orthodontist. Many studies have been performed with the objective of preventing the formation of white spot lesions and also to diagnose them as soon as possible.^1^ ^,^ ^2^ Among the most commonly used preventive methods, regular hygiene instruction, fluoridated toothpastes, fluoridated mouthrinses, fluoridated varnishes, adhesives containing fluoride, casein associated to amorphous calcium phosphate and nano-hydroxyapatite have been shown to be effective.^3^ ^,^ ^8^ ^-^ ^12^ Nano-hydroxyapatite is considered one of the most biocompatible and bioactive materials, due to the increased nanoparticles superficial area, which may facilitate the availability of the material and the reorganization of calcium phosphate ions in the form of hydroxyapatite.^14^ ^-^ ^16^ Besides, it’s association to fluorides may potentiate its effect, inhibiting demineralization and stimulating remineralization. Several studies have shown positive results with the use of nano-hydroxyapatite in the remineralization of caries lesions.^12^ ^-^ ^14^ However, there are no studies testing the effectiveness of fluoride associate to nano-hydroxyapatite in the prevention of demineralization around orthodontic brackets. 

Therefore, the objective of this study was to assess, *in situ*, the effect of a product containing fluoride associated to nano-hydroxyapatite, for the prevention of demineralization of the enamel adjacent to orthodontic brackets.

## MATERIAL AND METHODS

**Figure 1:**
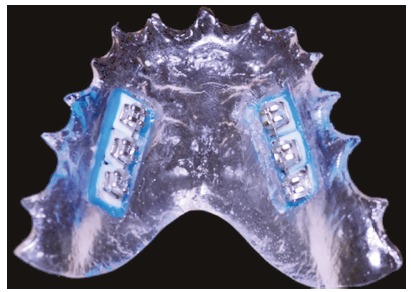
Palatal device with brackets bonded to the bovine enamel blocks and connected with 0.016-in NiTi wire.

The study was approved by the Institutional Review Board of the State University of Maringá, Brazil. This *in situ* study involved a randomized design performed in a period of 28 days (two stages of 14 days each), during which eight volunteers wore a palatal intraoral device containing six specimens of bovine enamel with orthodontic brackets bonded to them (Fig 1). Acording to Gameiro et al,^15^ a sample size of 30 enamel blocks per group is necessary for 80% power test in such microhardness *in situ* study. The inclusion criteria for the volunteers were: dental students, aged 20-25 years, without use of antibiotics or any other medication that would reduce saliva flow, and free from active caries lesions.

Bovine lower incisors from Nellore cattle, aged around 5 years, were extracted and immediately stored in 0.1% aqueous thymol solution. Teeth were cleaned and those with enamel stains, cracks and hypoplasia were discarded. They were sectioned (Isomet 1000; Buehler, Lake Bluff, IL, USA) to obtain one block per tooth measuring 5*x*5*x*2 mm, from the flattest area of the buccal area. They were sterilized in a 2% formaldehyde solution for a month. They were then polished (Arotec, São Paulo, SP, Brazil) using #320 sandpaper (1 min), followed by #600 and @1200 grit for 2 min each, at low speed. Polishing was completed with felt added with a diamond suspension of 1 µm, at high speed. The blocks were left immersed into deionized water for 12 h, to remove impurities. 

The enamel blocks fixed to acrylic disks were taken to a microdurometer (Shimadzu HMV2000, Tokyo, Japan) to measure surface microhardness. Three indentations were conducted at the center of each block at a distance of 100 µm between them with a Knoop indenter (50g for 5s). The average of the three indentations was calculated to obtain the average microhardness value. Enamel blocks presenting microhardness below 200 KNH were discarded. Ninety-six blocks were selected, presenting an average microhardness of 303.80 KNH (SD = 47.33). Each one received a number and was randomly allocated to the two experimental groups.

The studied groups were as follows: Control group CG (n = 48 enamel blocks), cariogenic challenge for 14 days - and did not receive any product application; Group GNH (n = 48 enamel blocks), experimental group - cariogenic challenge for 14 days with fluoride + nano-hydroxyapatite application on day 1 and 7.

The removable palatal devices were fabricated with acrylic resin containing two cavities (17 mm*x*6 mm*x*4 mm) at each side of the device. The blocks were fixed with sculpture wax and assembled in such a way that the enamel surface stayed 1 mm below the surface of the device (Fig 1), to allow the accumulation of biofilm.^15^ ^-^ ^17^ Before being fixed to the device, the surfaces of the enamel blocks were conditioned with 37% orthophosphoric acid (Condac 37, FGM, Joinvile/SC, Brazil) for 15 s, rinsed for 15 s and dried for 10 s with jets of air.^15^ The adhesive system (FGM, Joinvile, SC, Brazil) was applied according to the manufacturers instructions, and incisive brackets (Morelli, SP, Brazil) were bonded to the center of the enamel blocks with composite resin (Transbond, 3M, Monrovia, CA, USA). Then, a 0.016-in NiTi wire (Morelli, SP, Brazil) was fitted to the slots of the three brackets on each side, and fixed with individuals elastic modules (Morelli, Sorocaba, SP, Brazil)^15^ (Fig. 1).

The tested product (Desensibilize Nano-P, FGM, Joinvile, SC, Brazil) contains the following active ingredients: sodium fluoride (9000 ppm), nano-calcium phosphate (in the form of hydroxyapatite), and potassium nitrate; and as inactive ingredients: distilled water, thickener, surfactant, humectant, flavor, sweetener, and preservative.

The volunteers were instructed to wear the palatal devices for 14 consecutive days, make an interval of 7 days, and use them for another consecutive 14 days. The palatal device should be used continuously (24 h/day), except during meals and when performing normal oral hygiene after meals (breakfast, lunch and dinner). All volunteers received toothpaste without fluoride (Bitufo, Itupeva, SP, Brazil), soft toothbrushes (Bitufo, Itupeva, SP, Brazil), deionized water, gauze and 20% sucrose solution. They were instructed to brush their teeth three times daily but not to brush the enamel blocks. The volunteers were also instructed to remove the palatal device and drip 20% sucrose solution on each enamel block eight times daily.^15^ ^-^ ^16^ They were recommended not to eat or drink, except water, with the devices inside the mouth. 

During the first 14-day period, blocks received the treatment with fluoride + nano-hydroxyapatite, applied twice with the assistance of a microbrush on day 1 and 7 (group GNH). During the second period of 14 days, the enamel blocks did not receive any additional treatment (group CG). The same general instructions were repeated concerning the use, hygiene and the application of sucrose. After *in situ* period, the enamel blocks were repositioned onto their respective numbered acrylic disks and stored in plastic recipients moistened with absorbent paper and deionized water.

Enamel blocks were then longitudinally sectioned with a diamond disk assembled onto an electric sectioning machine. Half of the sample was submitted to the microhardness test, while the other half had the lesion depths analyzed by polarized light microscopy.

**Figure 2:**
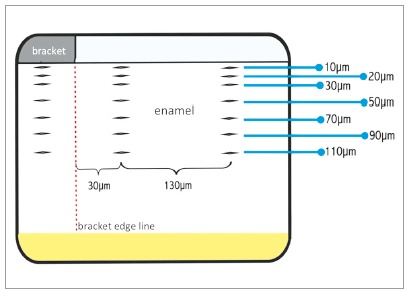
Illustration of the indentation lines (from 10 µm to 110 µm) under the bracket, 30 µm from the edge of the bracket, and 130 µm from the edge of the bracket.

For the longitudinal microhardness evaluation, the enamel blocks were embedded into 5 g of acrylic resin, using a metallographic embedder (under 150 Kgf/cm^2^ of pressure). With the longitudinal sectioning plane facing the resin surface, the samples were submitted to a surface polishing. Then longitudinal microhardness was evaluated with a Knoop indenter with a load of 25 g per 5s, making indentations lines at the depths of 10 µm, 20 µm, 30 µm, 50 µm, 70 µm, 90 µm, and 110 µm under the bracket base, and at 30 µm and 130 µm from the edge of the bracket (Fig 2).

**Figure 3:**
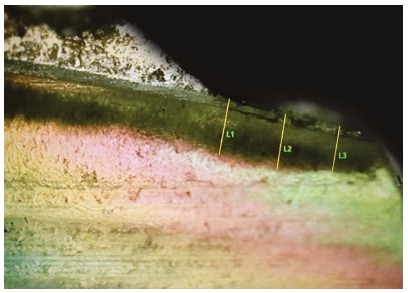
Reference lines (L1, L2 and L3) used for measuring lesions depth: L1 was taken from the bracket edge, L2 was taken at 150 µm, and L3 was taken at 300 µm from the edge.

For the polarized light microscopy evaluation, blocks were polished until reaching approximately 100 µm thick, and then they were assessed with polarized light microscopy at a magnification of 40*x*, to assess the lesion depth around the brackets. The cuts were observed under an Olympus BX50 microscope equipped with a 3CCD Pró-Series digital camera. Images were captured and analyzed by Image Pro-Plus (v. 4.5.1. Media Cybernetics). The photographs were taken with maximum illumination, and the depth of each lesion was measured within an distance of 300 µm from the bracket edge. Three readings were taken (at the bracket line, and at 150 µm and 300 µm from the edge) and the average of three measurements was calculated (Fig 3).

ANOVA and Tukey post-test were used for intragroup comparisons, and *t* tests were used for intergroup comparisons, at 5% of significance, using SPSS software v. 13.0 (Chicago. IL, USA).

## RESULTS

### Longitudinal microhardness

Inter and intragroup comparisons at the different microhardness depth measurements are presented in Tables 1 and 2 (under the bracket), 3 and 4 (30 µm from the bracket edge) and 5 and 6 (130 µm from the bracket edge). The main differences between groups occurred at 30 µm (at the depths of 10 and 20 µm) and at 130 µm (up to the depth of 70 µm) from the bracket edge, demonstrating significantly higher microhardness for the group GNH when compared to the CG (Tables 3 and 5). 

**Table 1:** Comparison between groups at different depths, under the brackets.

Depth	CG (n=48)	GNH (n=48)
10 µm	268.46 ± 71.48 KNH ^a^	305.33 ± 55.09 KNH ^a^
20 µm	315.68 ± 73.21 KNH ^a^	355.25 ± 55.16 KNH ^a^
30 µm	323.22 ± 70.63 KNH ^a^	374.29 ± 70.52 KNH ^b^
50 µm	345.25 ± 62.05 KNH ^a^	391.85 ± 56.92 KNH ^b^
70 µm	342.50 ± 61.50 KNH ^a^	392.50 ± 45.81 KNH ^b^
90 µm	349.79 ± 61.52 KNH ^a^	397.62 ± 50.76 KNH ^b^
110 µm	347.83 ± 69.81 KNH ^a^	393.64 ± 58.89 KNH ^a^

CG= Control Group, GNH= Experimental Group. Means followed by different lowercase letters in the same line represent statistically significant differences (ANOVA/TuKey; p < 0.05).

**Table 2:** Intragroup comparisons at different depths, under the brackets.

Depth	CG (n=48)	GNH (n=48)
10 µm	268.46 ± 71.48 KNH ^A^	305.33 ± 55.09 KNH ^A^
20 µm	315.68 ± 73.21 KNH ^AB^	355.25 ± 55.16 KNH ^B^
30 µm	323.22 ± 70.63 KNH ^B^	374.29 ± 70.52 KNH ^C^
50 µm	345.25 ± 62.05 KNH ^BC^	391.85 ± 56.92 KNH ^C^
70 µm	342.50 ± 61.50 KNH ^C^	392.50 ± 45.81 KNH ^C^
90 µm	349.79 ± 61.52 KNH ^BC^	397.62 ± 50.76 KNH ^C^
110 µm	347.83 ± 69.81 KNH ^C^	393.64 ± 58.89 KNH ^C^

CG= Control Group, GNH= Experimental Group. Means followed by different uppercase letters in the same column (intragroup) represent statistically significant differences (ANOVA/TuKey; p < 0.05).

**Table 3:** Comparison between groups at different depths, 30 µm from the edge of the bracket.

Depth	CG (n=48)	GNH (n=48)
10 µm	228.30 ± 98.21 KNH ^a^	315.56 ± 77.67 KNH ^b^
20 µm	284.31 ± 70.77 KNH ^a^	370.93 ± 79.38 KNH ^b^
30 µm	325.68 ± 78.27 KNH ^a^	387.50 ± 78.17 KNH ^b^
50 µm	354.33 ± 72.39 KNH ^a^	394.83 ± 66.81 KNH ^a^
70 µm	344.68 ± 69.00 KNH ^a^	388.60 ± 64.88 KNH ^b^
90 µm	350.81 ± 67.42 KNH ^a^	398.04 ± 55.08 KNH ^b^
110 µm	408.81 ± 420.95 KNH ^a^	401.70 ± 62.78 KNH ^a^

CG= Control Group, GNH= Experimental Group. Means followed by different lowercase letters in the same line represent statistically significant differences (ANOVA/TuKey; p < 0.05).

**Table 4:** Intragroup comparisons at different depths, 30 µm from the edge of the bracket.

Depth	CG (n=48)	GNH (n=48)
10 µm	228.30 ± 98.21 KNH ^AB^	315.56 ± 77.67 KNH ^A^
20 µm	284.31 ± 70.77 KNH ^B^	370.93 ± 79.38 KNH ^B^
30 µm	325.68 ± 78.27 KNH ^BC^	387.50 ± 78.17 KNH ^BC^
50 µm	354.33 ± 72.39 KNH ^BC^	394.83 ± 66.81 KNH ^BC^
70 µm	344.68 ± 69.00 KNH ^BC^	388.60 ± 64.88 KNH ^BC^
90 µm	350.81 ± 67.42 KNH ^BC^	398.04 ± 55.08 KNH ^BC^
110 µm	408.81 ± 420.95 KNH ^BC^	401.70 ± 62.78 KNH ^C^

CG= Control Group, GNH= Experimental Group. Means followed by different uppercase letters in the same column (intragroup) represent statistically significant differences (ANOVA/TuKey; p < 0.05).

**Table 5:** Comparison between groups at the different depths, 130 µm from the edge of the bracket.

Depth	CG (n=48)	GNH (n=48)
10 µm	216.30 ± 94.17 KNH ^a^	324.19 ± 91.53 KNH ^b^
20 µm	280.76 ± 84.35 KNH ^a^	359.66 ± 83.55 KNH ^b^
30 µm	297.60 ± 85.31 KNH ^a^	395.87 ± 70.65 KNH ^b^
50 µm	354.87 ± 68.72 KNH ^a^	408.87 ± 52.46 KNH ^b^
70 µm	353.45 ± 67.79 KNH ^a^	400.41 ± 61.48 KNH ^b^
90 µm	360.54 ± 75.10 KNH ^a^	405.97 ± 53.11 KNH ^b^
110 µm	354.62 ± 70.03 KNH ^a^	407.18 ± 58.30 KNH ^b^

CG= Control Group, GNH= Experimental Group. Means followed by different lowercase letters in the same line represent statistically significant differences (ANOVA/TuKey; p<0.05).

For the intragroup comparisons, it was found that the microhardness presented main significant differences until 30 µm of depth (Tables 2, 4 and 6).

**Table 6:** Intragroup comparisons at the different depths, 130 µm from the edge of the bracket.

Depth	CG (n=48)	GNH (n=48)
10 µm	216.30 ± 94.17 KNH ^A^	324.19 91.53 KNH ^A^
20 µm	280.76 ± 84.35 KNH ^A^	359.66± 83.55 KNH ^A^
30 µm	297.60 ± 85.31 KNH ^BC^	395.87 ± 70.65 KNH ^B^
50 µm	354.87 ± 68.72 KNH ^C^	408.87 ± 52.46 KNH ^B^
70 µm	353.45 ± 67.79 KNH ^C^	400.41 ± 61.48 KNH ^B^
90 µm	360.54 ± 75.10 KNH ^C^	405.97 ± 53.11 KNH ^B^
110 µm	354.62 ± 70.03 KNH ^C^	407.18 ± 58.30 KNH ^B^

CG= Control Group, GNH= Experimental Group. Means followed by different uppercase letters in the same column (intragroup) represent statistically significant differences (ANOVA/TuKey; p<0.05).

### Polarized light microscopy

One specimen from CG and four from GNH broke during enamel cut preparation for the microscopy evaluation. Then 47 enamel slices from CG and 44 from GNH were examined under polarized microscopy. 

**Figure 4:**
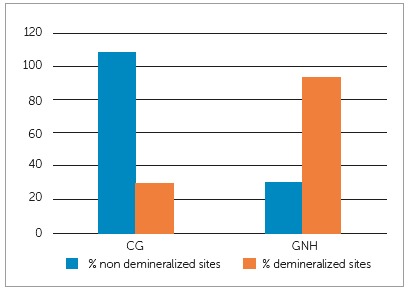
Graph showing percentage distribution of sites assessed with and without demineralization, under polarized light microscopy exam.

Table 7 illustrates the intergroup comparisons of mean demineralization depths, showing significant reduction in mean depth in GNH group. Among sites examined (3 reference points in each cut), demineralization areas were found in 78% (110/141) in the control group (CG) and 28% (37/132) in the experimental group (GNH) (Fig 4). 

**Table 7:** Comparison of the mean demineralization depths between groups

Groups	Mean (µm)	Standard Deviation
CG (n=47)	76.43^a^	83.75
GNH (n=44)	15.01^b^	33.65

CG= Control Group, GNH= Experimental Group. Different letters correspond to statistically significant differences (p<0.05).

## DISCUSSION

Enamel demineralization around orthodontic appliances continues to be a common clinical problem, even in face of the application of fluoridated materials, which minimize but do not solve the problem completely.^1^ ^-^ ^3^ ^,^ ^7^ ^,^ ^8^ The present study tested *in situ* a product containing fluoride and nano-hydroxyapatite that reduced demineralization around brackets (Tables 1, 3, 5 and 7), confirming fluoride role on such protection. 

The *in situ* model with intraoral devices has been frequently used, as it simulates the caries process that occurs within the oral cavity. Ideally, all the dental caries etiological factors such as dental substrate, diet, formation of biofilm and time should be involved.^17^ ^-^ ^19^ The main advantages of this type of study, according to Zero,^19^ are the performance of the study within the human oral cavity; flexibility of the experimental model; variables easily controllable; short operational time; and less cost when compared to longitudinal clinical studies. The model adopted in the present study followed the modifications suggested by Gameiro et al.^15^, which includes brackets bonded to the enamel blocks. 

The group that received fluoride + nano-hydroxyapatite demonstrated lesions significantly reduced under polarized optical microscopy (Table 7), as well as reduced demineralization assessed by microhardness (Table 3 and 5). This result suggests a positive effect of the tested product. Besides the well known effectiveness of fluoride, nano-hydroxyapatite may have contributed to the mineralization of the outer layer of white spot lesions by depositing nanoparticles of apatite in the defects of demineralized enamel.^12^ ^,^ ^21^ ^-^ ^22^ Moreover, nano-hydroxyapatite can act by providing a source of calcium for the oral cavity, increasing its levels and leading to a limitation to the acid challenge, reducing enamel demineralization while promoting its remineralization. This reservoir of calcium phosphate may help a state of oversaturation concerning enamel minerals and, thus, decreasing demineralization and potentiating remineralization.^14^ ^,^ ^24^ ^-^ ^25^ It was reported that nano-hydroxyapatite promotes remineralization preferably on the superficial layer of the enamel lesion.^14^ However, this process was shown to be not feasible under neutral conditions, while under acid conditions it can significantly accelerate remineralization rate, depth and the extension of incipient lesions. This is in agreement with the findings in this work. In cariogenic conditions (dripping of 20% sucrose 8x/daily), nano-hydroxyapatite and fluoride demonstrated to be effective in the protection against enamel demineralization, corroborating with the literature.^14^ ^,^ ^23^ ^,^ ^25^

Many studies reported from 40 to 50% reduction in demineralization around brackets under fluoride or hidroxiapatite-containing products applications. This seems to be less preventive effect than we found from the combination of both. Unfortunately, our study did not present a nano-hydroxiapatite nor a fluoride exclusive groups to isolate its effects from their association.

Previous *in vitro* and *in vivo* studies have already demonstrated a demineralization preventive or reducing potential of fluoride-containing products.^3^ ^,^ ^7^ ^,^ ^8^ ^,^ ^21^ ^,^ ^22^ Other studies indicated that preventive programs that use daily fluoride mouthrinses^1^ ^,^ ^2^ may protect the orthodontic patient from white spots, but they are dependent on the cooperation of the patients to achieve such an objective. The topical application of varnishes containing 5000 ppm of fluoride is advantageous in this respect, as it only depends on the professional application, reducing the influence of patient cooperation on the results.^3^ ^,^ ^7^ ^,^ ^21^ On average, these methods have been shown to reduce between 40 to 50% of the incidence of white spot lesions around brackets. In the present stud,y GNH group showed 2.7 times less demineralization areas, when compared to CG (Fig 4). Such effect was confirmed when mean depth measures were compared (Table 7). 

Considering the limitations of the *in situ* study, fluoride associated with nano-hydroxyapatite *in situ* effects demonstrated relevant enamel protection, and inspires a future *in vivo* study to verify its behavior in orthodontic patients.

## CONCLUSION

Taking into consideration the methodology used and the data derived from the present study, it may be concluded that the tested product containing fluoride associated with nano-hydroxyapatite presented a preventive effect on the demineralization of enamel adjacent to orthodontic brackets, and it may be an alternative for the treatment of patients with high risk of caries. 
